# Optimal Human Functioning Requires Exercise Across the Lifespan: Mobility in a 1g Environment Is Intrinsic to the Integrity of Multiple Biological Systems

**DOI:** 10.3389/fphys.2020.00156

**Published:** 2020-02-27

**Authors:** David A. Hart, Ronald F. Zernicke

**Affiliations:** ^1^Faculty of Kinesiology, University of Calgary, Calgary, AB, Canada; ^2^McCaig Institute for Bone and Joint Health, University of Calgary, Calgary, AB, Canada; ^3^Department of Surgery, University of Calgary, Calgary, AB, Canada; ^4^Alberta Health Services, Bone and Joint Health Strategic Clinical Network, Edmonton, AB, Canada; ^5^Department of Orthopaedic Surgery, Michigan Medicine, University of Michigan, Ann Arbor, MI, United States; ^6^School of Kinesiology, University of Michigan, Ann Arbor, MI, United States; ^7^Department of Biomedical Engineering, University of Michigan, Ann Arbor, MI, United States; ^8^Exercise & Sport Science Initiative, University of Michigan, Ann Arbor, MI, United States; ^9^Department of Physiology and Pharmacology, University of Calgary, Calgary, AB, Canada

**Keywords:** exercise and health, mobility and optimal function, obesity, chronic disease, overuse injuries, musculoskeletal system, cognitive function, microgravity

## Abstract

It is widely acknowledged that achieving and maintaining a healthier lifestyle can be enhanced through regular participation in sport and physical activity. Coevally, a growing number of health professionals regard exercise as a legitimate intervention strategy for those who have lost their health. Exercise has been shown to be effective for overweight or obese individuals, who are at risk to lose their health due to development of type II diabetes, cardiovascular disease, as well as, infiltration of muscles, bone and other organs with fat, so it can be considered medicine. However, exercise and associated mobility likely also have a strong prevention component that can effectively contribute to the maintenance of the integrity of multiple biological systems for those who do not have overt risk factors or ongoing disease. While prevention is preferred over intervention in the context of disease, it is clear that exercise and associated mobility, generally, can be an effective influence, although overtraining and excessive loading can be deleterious to health. The basis for the generally positive influence of exercise likely lies in the fact that many of our physiological systems are designed to function in the mechanically dynamic and active 1g environment of Earth (e.g., muscles, cartilage, ligaments, tendons, bones, and cardiovascular system, and neuro-cognitive function), and nearly all these systems subscribe to the “use it or lose it” paradigm. This conclusion is supported by the changes observed over the more than 50 years of space flight and exposure to microgravity conditions. Therefore, the premise advanced is: “exercise is preventative for loss of health due to age-related decline in the integrity of several physiological systems via constant reinforcement of those systems, and thus, optimal levels of exercise and physical activity are endemic to, essential for, and intrinsic to optimal health and wellbeing.”

## Introduction

Arguably, exercise and its associated mobility are integral to optimal health and wellbeing of humans. This statement generates questions related to how does it work, which biological and physiological systems benefit the most from its effects, and why? Is it important to know these answers? The concept that “Exercise is Health” existed for centuries (Hills et al., 2015), and events of the past 50 years related to multi-national space programs now provide unique potential clues to as to why exercise is so effective from the perspectives of both prevention and intervention.

Undoubtedly, exercise (aerobic and/or resistance) can be medicinal for those with diagnosed disease conditions. For example, for some individuals, exercise may alter symptoms or progression of diabetes (Way et al., 2016), dementia or loss of cognition (Liu-Ambrose and Donaldson, 2009; Ma et al., 2016), loss of cardiovascular integrity (Ribeiro et al., 2016), impaired bone formation and rate of loss in post-menopausal females (Moreira et al., 2014), osteopenia in general (Scott et al., 2008), and muscle atrophy (Carter et al., 2015), to name several of the most prevalent. However, once a disease has been established, the effectiveness of any intervention (e.g., exercise) may be diminished as the disease process implies dysfunction of normal homeostatic control; exercise is not a cure for the condition but part of the total intervention strategy (e.g., drugs, surgery, and exercise). In this context, exercise is likely medicinal but is not the whole picture. Prevention is a preferred approach versus intervention after the fact.

The reasons why exercise in the context of disease may be less effective than in the absence of disease are varied, in part due to the chronicity of diseases where it is reported to be effective, and in part due to the pharmacological interventions used. Firstly, disease implies pathology, and pathology implies dysfunction in the target integrity. Secondly, many diseases are treated with pharmacological agents, which in themselves may impact the pathology and contain, but not cure the conditions. Thus, an exercise protocol, added to drug interventions, will have its impact influenced by both the disease and the drugs. Thirdly, in many chronic diseases, such as those in which exercise reportedly has influence, target tissues can undergo epigenetic modifications (Brunet and Berger, 2014; Raman et al., 2018; Sqalla et al., 2018), which alter their responsiveness to normal stimuli, drugs, and other interventions (Piletic and Kunei, 2016). These conditions include Alzheimer’s disease (Qazi et al., 2018), osteoporosis (Ghayor and Weber, 2016), osteoarthritis (Van Meurs, 2017), rheumatoid arthritis (Cribbs et al., 2015), cardiovascular disease (Ahuja et al., 2017; Ghosh et al., 2017), diabetes (Muka et al., 2016; Raghuraman et al., 2016), and sarcopenia (Sharples et al., 2016). Exercise itself may result in epigenetic alterations both before and after development of chronic disease development (Grazioli et al., 2017), but whether exercise alone is preventative for disease development will require more investigation. Thus, the distinction between “Exercise is Medicine” and “Exercise is Health” has validity, and may explain why exercise in a prevention mode is preferred over that in a medicinal role, but that distinction does not imply that “exercise is medicine” is not a valuable intervention (Sallis, 2009).

Exercise can also contribute to the health of individuals at risk for losing it, such as in obese individuals, via re-establishing metabolic control, caloric balance, and other related energy utilization parameters. As a consequence, exercise can impact multiple biological and physiological systems via metabolic control. Exercise can influence conditions such as type II diabetes onset and progression at the level of muscle-mediated insulin-resistance (Way et al., 2016), as well as aspects of the metabolic syndrome and chronic inflammation associated with obesity, and many other aspects of the condition (Pedersen and Saltin, 2015). Nevertheless, even with obesity, epigenetic modifications can occur and progress over time (Desiderio et al., 2016; Pigeyre et al., 2016; Xu et al., 2016), and such changes may have transgenerational influences in both humans (Bays and Scinta, 2015) and preclinical models (Paul et al., 2019), so treating early obesity rather than long-term chronic obesity with exercise may yield substantially different outcomes for many individuals.

As more is learned about epigenetic modifications of both somatic and germ-line cells, there may be opportunities to reverse their potential negative impact on gene regulation, as well as enhance the positive influences. However, such epigenetic changes may be associated with alterations to DNA methylation, histone modifications, and some RNA-mediated processes (reviewed in Ling and Ronn, 2019), and as such will require a detailed analysis of negative and positive alterations, and whether transgenerational influences are associated with the changes. Interestingly, exercise is known to lead to epigenetic alterations of both skeletal muscle (reviewed in Widmann et al., 2019) and in germ cells leading to transgenerational effects, particularly as related to metabolic pathways (Denham, 2018; Axsom and Libonati, 2019). In the review by Axsom and Libonati (2019), the focus was on DNA methylation changes in offspring and included studies of both preclinical models and humans. However, it should be noted that the number of epigenetic-related studies performed to date has not been large, the number of studies done on humans versus preclinical models is small for obvious reasons associated with invasiveness, the exact mechanisms via which the epigenetic changes occur are still lacking in detail, and it remains to be determined which types of exercise (e.g., aerobic versus resistive) are most effective for generating adaptations. While there are limitations to the current findings, they are encouraging regarding exercise and disease risks (i.e., heart disease and cancer) and the transgenerational influences of such modifications—the latter may be contributed by both parents (Ashe and Khan, 2004; Ahuja et al., 2017; Denham, 2018; Axsom and Libonati, 2019). Furthermore, some epigenetic changes occur during aging (Pal and Tyler, 2016; Pagiatakis et al., 2019), so the effectiveness of exercise at the epigenetic level may be influenced by the age that it is implemented and sustained—potentially a life-long chronic exercise routine will be best.

Several of the above conditions are associated with aging, and indeed, dementia, cardiovascular diseases, sarcopenia, and osteoporosis are more common in mature or elderly populations. Obesity, with unique fat deposition patterns in males and post-menopausal females, was in the past more common in older individuals, particularly those with mobility issues, but also those who became more sedentary after a life of hard physical labor. However, more recently, younger individuals with sedentary occupations or lifestyles, compounded by poor diets (e.g., high in saturated fats and sucrose), are becoming obese. Regrettably, children with obesity constitute one of the fastest growing populations (Karnik and Kanekar, 2012), which generates concern for multiple reasons that include early onset of type II diabetes and cardiovascular disease, as well as musculoskeletal concerns related to growth and maturation. With obesity comes infiltration of muscle and bone with fat, which can have biomechanical consequences during post-puberty maturation of the skeleton articular system. Childhood or adolescent obesity effects on bone-muscle integrative functioning may remain altered even if weight is lost as an adult. Concomitantly, these effects may be related to epigenetic modifications, which can interfere with the effectiveness of exercise. Thus, exercise, tailored to the individual, has the potential to mitigate the risk for development of several conditions prior to their onset in individuals with obesity. In rats, exercise has been shown to be preventative for the consequences of diet-induced obesity (see Collins et al., 2016 for diet details) when implemented at the same time as the animals were started on such a high-fat and sugar diet (Rios et al., 2019).

The above-discussed progression leads to a third important function of exercise: the preventative aspects throughout the lifespan to maintain the integrity of multiple physiological systems including muscle, bone, cardiovascular, and respiratory systems, as well as brain function. It is generally recommended that adults and children engage in about 150 min of moderate exercise per week. Engaging in such exercise, at a minimum, throughout the lifespan has many benefits based on the literature. In addition, different types of exercise (e.g., aerobic vs. resistance) can have differential effects on tissue adaptation and quality. For example, Armamento-Villareal et al. (2019) reported how some forms of exercise can be more effective at protecting against bone-loss during weight-loss therapy in older adults with obesity. With similar amounts of weight loss, elderly individuals (≥65 years) who combined aerobic and resistance exercises or only resistance exercises during their weight-loss program had significantly less weight loss-induced decreases in bone mineral density versus those who only participated in aerobic exercises.

**FIGURE 1:**
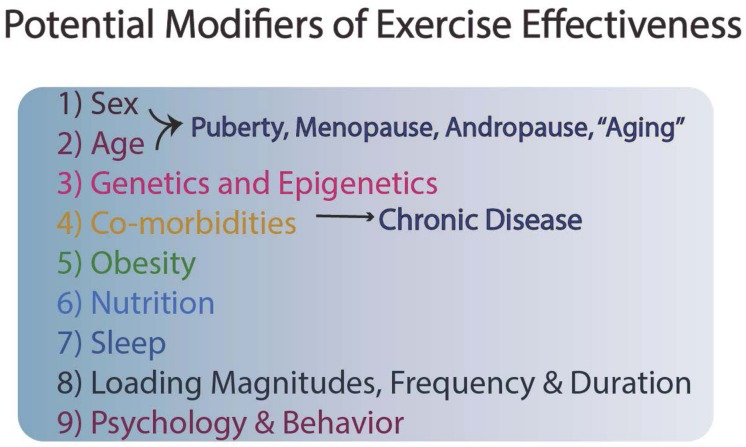
Potential Modifiers of Exercise Effectiveness.

Although exercise and enhanced mobility are cost-effective prevention strategies in which nearly all individuals can engage, undoubtedly genetics also plays a role in risk for cardiovascular disease, osteoporosis, and other conditions, so exercise is only one component of a series of modifiable activities for an individual (Figure 1). Often exercise has to be optimally matched with good dietary habits, sufficient sleep, and socialization to reap optimal benefits (Figure 1).

In terms of the positive effects of exercise on cognitive function, a growing body of evidence suggests that cognitive function can be enhanced via short-term and long-term exercise and physical activity. Won et al. (2019) studied brain activation during a semantic memory task after a single bout of exercise in healthy older adults (55–85 years) using functional magnetic resonance imaging (fMRI)—semantic memory includes things that are “common knowledge” (e.g., names of colors, sounds of letters, capitals of countries, and other basic facts acquired over a lifetime). On separate days, subjects engaged in either 30 min of rest or 30 min of stationary cycling exercise immediately before performing a memory task during fMIR scanning. Acute exercise resulted in significantly greater semantic memory activation in the middle frontal, inferior temporal, middle temporal, and fusiform gyrus, as well as greater activation in the bilateral hippocampus. Complementing those positive responses, there are numerous studies that highlight the positive effects of longer-term exercise on cognitive and neural plasticity in older adults. In a comprehensive and critical review of related research, Kramer et al. (1999) and Erickson and Kramer (2009) concluded that even 6 months of moderate levels of aerobic activity are sufficient to produce significant improvements in cognitive function, particularly on measures of executive function. Colcombe and Kramer (2003) also reported that exercise broadly improved cognitive function across a number of domains including spatial functioning and executive control. The positive effects of exercise on cognitive function appear to extend to older adults with dementia; Heyn et al. (2004) reported that aerobic exercise interventions could reliably reverse cognitive impairments in demented individuals. In like manner, patients in the early stages of Alzheimer’s disease who were more aerobically fit had less whole-brain atrophy and white matter atrophy than those patients who were less aerobically fit (Burns et al., 2008). In summarizing the state-of-knowledge about the effects of exercise on brain function, Erickson and Kramer (2009) posited that “…an active lifestyle with moderate amounts of aerobic activity will likely improve cognitive and brain function and reverse neural decay frequently observed in older adults.” (p. 24). That conclusion by Erickson and Kramer (2009) was strongly corroborated by a Norwegian population-based prospective study (>30,000 men and women participants) that assessed the temporal changes in cardiorespiratory fitness and risk of dementia incidence and mortality (Tari et al., 2019). Tari et al. (2019) found that individuals who sustained high estimated cardiorespiratory fitness had a reduced risk of incident dementia and reduced risk of dementia mortality. Participants who increased their estimated cardiorespiratory fitness over time, gained 2.2 dementia-free years and 2.7 years of life when compared to those who remained unfit. Complementing the positive effects of aerobic exercise on cognitive function, Liu-Ambrose and Donaldson (2009) reported that incorporating resistance training, in conjunction with aerobic exercise, among senior individuals can also produce cognitive benefits.

While the positive effects of exercise permeate across a host of biological systems, over-exercising and excessive tissue loading can lead to deleterious effects and injury. The same parameters of exercise (i.e., duration, frequency, and intensity) that influence positive responses can also influence the risk of injury (Jones et al., 1994). Catastrophic failures of tissues (e.g., tendons, ligaments, and bone) occur when the ultimate loads of a tissue are exceeded. Such catastrophic injuries (e.g., patellar tendon rupture) have been reported for activities such as Olympic weightlifting (Zernicke et al., 1977), and extensive epidemiological data document the pervasive rates of knee anterior cruciate ligament (ACL) injuries in athletes (Joseph et al., 2013). ACL injuries account for over half of all knee injuries in sport (Risberg et al., 2004). In an extensive analysis of United States high school students participating in athletics—with nearly 10 million athlete-exposures during 2007–2012, Joseph et al. (2013) found that participants were seven times more likely to sustain an ACL injury in competition than in practice, and the highest ACL injury rates occurred in girls’ soccer (12%) and boys’ American football (11%), with the lowest rate in baseball (<1%).

Excess training and overuse can also produce significant injuries in bone (i.e., stress reactions and stress fractures). In particular, long-distance runners and military personnel are susceptible to bone stress injuries. Significant epidemiological data have been collected in both the Israel and United States armies on bone stress fractures of male and female soldiers (Friedl et al., 2008), with the data revealing 50% of women have one or more injuries by the end of basic training, including stress fractures. The high rates of exercise-related stress fractures in women—and also in men—can be linked to components of the Female Athlete Triad: (1) low energy availability with or without disordered eating, (2) menstrual dysfunction (females), and (3) low bone mineral density. Individuals can present with one or more of these Triad components (DeSouza et al., 2014). For male distance runners, Kraus et al. (2019) recently published a 7-year retrospective and prospective study of the bone stress injury rates of 156 male United States collegiate distance runners. They used applicable risk assessment categories from a “modified” Female Athlete Triad and discovered that male runners presented with risk factors for bone stress injuries that paralleled those found in female runners as described by the Female Athlete Triad, including “…low energy availability, low body mass index, prior bone stress injury, and low bone mineral density values.” (p. 237). Their results highlighted the importance of optimizing nutrition and energy availability and achieving optimal body mass index to sustain and enhance skeletal health.

Recent data (Tenforde et al., 2016) revealed new insights about the relation between Female Athlete Triad Risk Assessment Stratification and the development of bone stress injuries. Tenforde et al. (2018) classified female collegiate athletes in 16 sports into low-, moderate-, and high-risk categories using the Female Athlete Triad Risk Assessment score and used those scores to compare incidence of bone stress injuries. Sports with the highest proportion of moderate- and high-risk scores included gymnastics, lacrosse, and cross-country runners, with the cross-country runners sustaining the majority of the bone stress injuries. To complement those results, Tenforde and researchers at Stanford University, Harvard University, and the University of North Carolina collectively investigated the interrelations among Triad Risk factors, sport-specific loading factors, and bone mineral density in female collegiate athletes. They analyzed data of 239 athletes across 16 different sports with a range of loading characteristics (e.g., high-impact and multidirectional vs. non-impact sports). They discovered that both sport type and Triad risk factors affected bone mineral density. Synchronized swimmers and swimmers/divers had the lowest bone mineral densities, while the highest bone mineral densities were found in gymnastics, volleyball, and basketball athletes. Athletes in non- or low-impact sports, with low body mass index, and oligomenorrhea/amenorrhea were at highest risk for reduced bone mineral density.

One of the lingering questions regarding the systems affected is whether some of these responses are interrelated (e.g., improved blood flow and cardiovascular system function associated with enhanced brain function), or the responses could reflect independent target systems that are uniquely sensitive to the mechanical stimulation associated with specific exercise regimens. Another lingering question relates to “precision health”—analyzing and integrating complex, individual-specific genetic, physiological, and environmental data to sustain health and well-being and to predict and prevent disease or optimize individual treatment and interventions. There is no doubt that different people respond to different exercise strategies. Healthy individuals vs. individuals with cardiovascular, pulmonary, and/or diabetes diseases may have varying responses to training modalities (moderate-intensity continuous exercise training vs. high-intensity interval training) (Ross et al., 2016). How specific exercise interventions and training can be personalized to each specific individual remains as a challenge to address with future research.

To achieve the above-mentioned goal will require more information and understanding regarding the mechanisms involved in responsiveness to exercise and better information regarding factors that modulate such responsiveness. There is clear evidence that sex can play a role in responsiveness to exercise at the level of the brain, with females responding more than males (reviewed in Barha et al., 2017, 2019; Dao et al., 2019). This is an interesting response pattern since most Alzheimer patients are female. Furthermore, it has been reported that some people are responders and others non-responders to both aerobic exercise and resistance training (reviewed in Sparks, 2017). In addition, the type of exercise may be critical. Most studies have focused on aerobic exercise, but the type of exercise may be important in how a target system is affected, such as the cardiovascular system (Barha et al., 2017). This latter point implies that some of the mechanisms involved can vary, and there may not be a single molecular explanation for the effectiveness of exercise.

**FIGURE 2:**
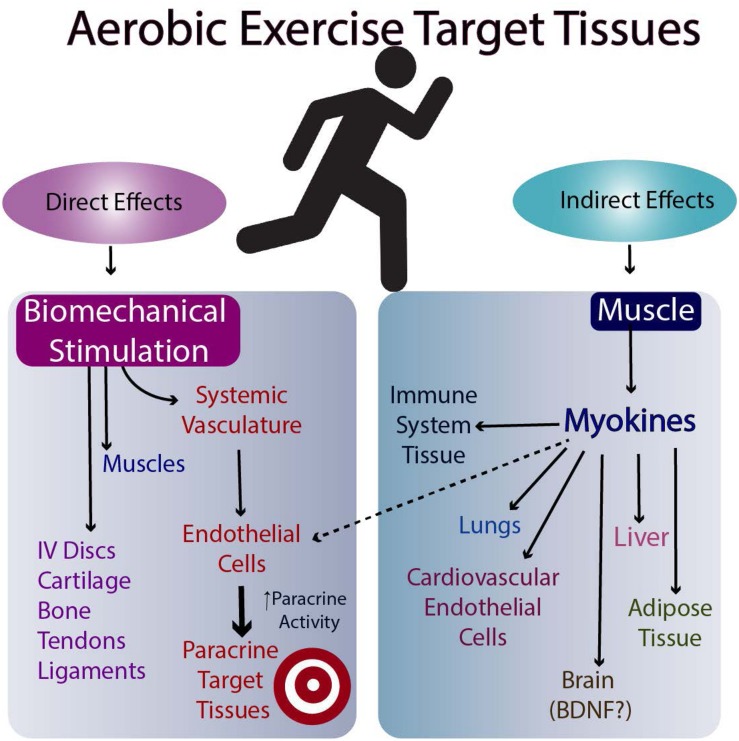
Possible Mechanisms by which Aerobic Exercise Impacts Multiple Target Tissues.

**FIGURE 3:**
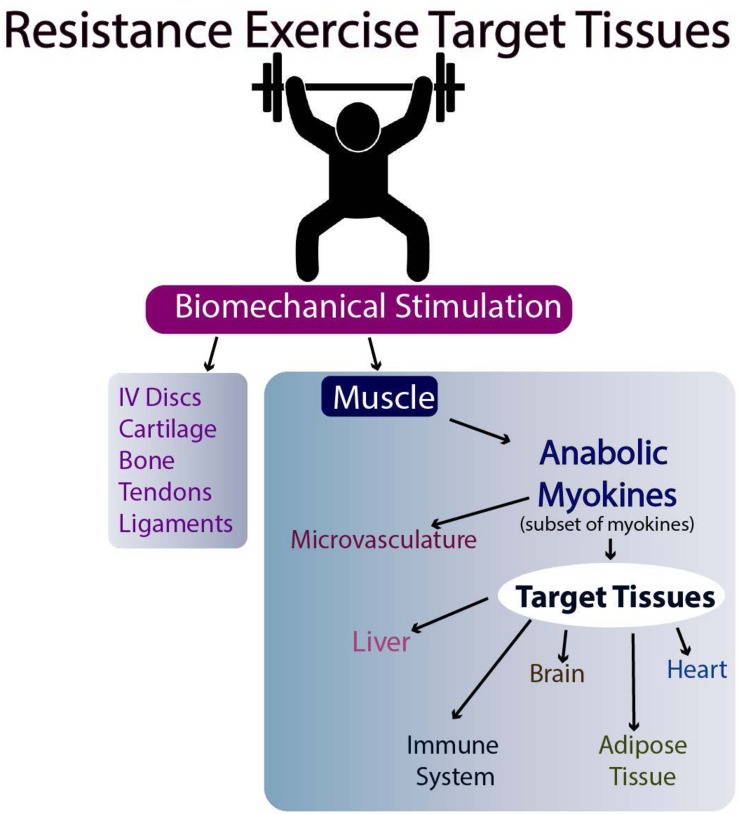
Resistance Exercise and Effectiveness on Target Tissues.

The molecular basis for the effectiveness of exercise in the brain is believed to relate to the brain levels of mature BDNF (brain-derived neurotrophic factor), but the results in humans are equivocal (discussed in Barha et al., 2017). Another possibility is the stimulation of myokine release from skeletal muscle following exercise (Nielsen and Pedersen, 2008, Nielsen and Pedersen, 2011, 2017; Pedersen and Febbraio, 2012; Giudice and Taylor, 2017; Eckel, 2019). Such myokines can then travel to target tissues via the circulation and affect their metabolism (Figures 2, 3). Whether they affect the target tissues directly or indirectly via the microvasculature in different tissues remains to be determined. Finally, exercise may directly influence the metabolism of tissues responsive to biomechanical signals via the loading associated with the exercise (i.e., muscle, tendons, bone, ligaments, and endothelial cells of the microvasculature). Some of these tissues respond to tensile and compressive loading, while endothelial cells of the microvasculture can respond to stress-related changes associated with changes in blood flow with exercise (Ando and Kamiya, 1996; Boisseau, 2005). Some of these potential interrelations are outlined in Figures 2, 3.

From the above discussion, some clues regarding the mechanisms involved in how exercise is effective have been surmised, but many questions still remain unanswered. The answers to these and related questions are difficult to untangle in populations who are restricted to living on Earth.

## Mobility and Navigation Through the Environment

From the preceding discussion, it is clear that mobility and mechanical loading of multiple tissues are likely intrinsic aspects of human evolution and progression to current *Homo sapiens*. However, mobility is only one aspect of functioning, with mobility and navigation (patterned mobility) integrated to contribute to survival (e.g., evading predators and hunting) and performing a multitude of tasks. Thus, navigation requires integration of mobility, vision, and neural control (brain) involvement (reviewed in Hart et al., 2019). Visual compromise with aging (e.g., cataracts, macular degeneration, or diabetic retinopathy) can inhibit function even if mobility is maintained and sustained. In addition, navigation involves other senses (e.g., hearing, smell, and touch). Recently, it was reported that diet-induced obesity in a preclinical model led to eye changes (Collins et al., 2018), and those changes could be affected, in part, by exercise. In addition, there also appears to be a link between compromise in knee integrity and the eye in other circumstances such as uveitis in juvenile idiopathic arthritis and additional links between the eye and the knee as evidenced by changes in the eye following a knee injury in preclinical models (Kydd et al., 2007); discussed in Hart, 2018b). Thus, there are multiple ways to interfere with optimal functioning related to mobility and navigation compromises due to loss of integration, which is essential for effective navigation of the organism or person.

## Building on Information From Spaceflight-Associated Loss of System Integrity

Building on the premise that optimal physiological functioning on Earth requires exercise and mobility, we posit that important and creative insights can be gleaned from space programs where exposure to microgravity or microgravity analogs generates situations diametrically opposed to exercise (i.e., loss of system integrity). Humans were not designed for microgravity environments, but the adaptive responses of humans to space flight and microgravity have the potential to reveal unique insights about the integrated responses of physiological systems to physical activity versus inactivity and explanations for why exercise is both necessary and effective to maintain health.

An often ignored reality of life is that on Earth we live in a 1g environment. During evolution, at some point, the migration from an ocean or aquatic-based environment required developing the ability to function in a 1g environment with its associated ground reaction forces (Morey-Holton, 2003). For humans, this required developing systems that allowed for upright walking and running, requirements that required an enhanced cardiovascular system, modifications to the spinal column, enhanced muscle function, and adaptations for coordinated and interactive neuro-control mechanisms from the brain to the periphery. As *Homo sapiens* represent human adaptations to this point, such adaptations allow us to function effectively in this 1g environment (Morey-Holton, 2003). Further, the implication of our Earth-related 1g adaptation is that from fetal development onward, we have been prepared for and continuously adapt to this environment during childhood and adolescence. The 1g environment is inextricably linked to regulation during maturation and in maturity, and advancing age likely impacts patterns of senescence due to subtle or overt failures of system regulation.

With the onset of human space exploration nearly six decades ago, we are now afforded the opportunity to investigate the influence of prolonged microgravity on humans. Interestingly, several of the same systems positively influenced by exercise on Earth are negatively influenced by exposure to microgravity (e.g., muscle atrophy with fatty infiltration, loss of bone quality and structure, loss of cardiovascular tone, and altered features of cognition) (Zernicke et al., 1990; Grabherr and Mast, 2010; Ploutz-Snyder et al., 2014; Ohira et al., 2015; Grimm et al., 2016; Hughson et al., 2016; Hart, 2018a). These systems, which appear to be uniquely and ubiquitously sensitive to microgravity effects, may be sentinel systems of the human body that have most effectively adapted to functioning in a 1g environment; they adhere to the use it or lose it paradigm (Scott et al., 2008; Hart and Scott, 2012; Hart, 2018a). Based on current efficacy of space-based, countermeasure attempts to overcome the negative influences of microgravity, some of the systems (e.g., skeletal muscle atrophy) are more readily responsive than others (e.g., osteopenia). Similarly, maintenance of integrated system integrity on Earth in the 1g environment is likely not based on multiple completely independent systems, but operate as highly integrated and interactive elements, with—as yet—unidentified central regulatory components. Even with chronic exposure to the 1g environment on Earth, the function and responsiveness of many of these same systems decline with age—often independently from each other. Thus, if there is a “central regulator” of these systems, some downstream components may decline with age independently—possibly due to otherwise silent mutations or epigenetic alterations. Furthermore, the regulation of these systems on Earth or in space may be sex-dependent, as several are influenced by hormones, and it is clear that regulation in females can vary across the lifespan due to events such as pregnancy and menopause. Thus, as astronauts are usually fit and free of overt chronic diseases of the systems affected by space flight, the findings of the changes associated with exposure to microgravity strongly support the notion that “Exercise is Health” and essential to maintain the integrity of the human system which evolved in the 1g environment of Earth.

## The “Use It or Lose It” Paradigm

Even on Earth, the use it or lose it paradigm is evident. If a leg is immobilized in a cast, the muscles of that lower extremity will atrophy. Once the cast is removed and the limb is re-mobilized, the affected muscles re-adapt to a new set point. With prolonged bed rest, muscles atrophy and can become infiltrated with fat (Trudel et al., 2009). Similarly, bones can lose their integrity with prolonged bedrest, and that skeletal loss can be rapidly initiated (Kos et al., 2014). Relatedly, with bedrest, cardiovascular tone is altered, possibly involving neural control elements in ways resembling what happens in space (Hughson and Shoemaker, 2015). Thus, disuse atrophy occurs on Earth in some of the same systems as are observed to be altered in space and following exposure to microgravity.

The questions then arise as to why and how such changes occur in space and on Earth. Related to induction of atrophy, the menisci of the rabbit knee have been used as a model to better understand the involved mechanisms (Natsu-ume et al., 2005). Removing the menisci from the knee led to the rapid de-repression of a cascade of catabolic genes that could be detected within 4 h of removing the tissue from the animal. Applying intermittent cyclic compressive loading, analogous to what the tissue experiences *in vivo*, prevented the de-repression of this catabolic set of genes. Thus, a set of catabolic genes that can degrade a specific tissue were held in check by exogenous loading, such as that conveyed by ground reaction forces. Potentially, similar mechanisms apply to other tissues as well, but the genes involved may differ somewhat with different tissues; those specific differences, however, remain to be elucidated.

Conversely, loading of a tissue can also lead to up-regulation of genes that contribute to tissue integrity or healing processes. Different types of loading may be applied to a tissue (e.g., compression, shear, or tension), and cell responses can be loading-type specific (Majima et al., 2000). Vascular endothelial cells experience shear loading due to blood flow and the pulsatile nature of shear promoted by arterial forces (Koida and Hambrecht, 2005; Johnson et al., 2011). Endothelial cells experiencing loading respond to the stimuli by enhancing or suppressing production of specific molecules, and such responses influence endothelial cell functioning. Therefore, strong evidence exists at the target cell level for the how exercise and biomechanical loading may impact cells.

As noted, in space both bone and muscle can undergo atrophy and current mechanical countermeasures have proven to be reasonably effective in preventing muscle atrophy, but not as effective for bone loss. Interestingly, loss of bone while in space is more evident for bones of the lower extremities than the upper extremities (Hart, 2018a)—which are used extensively for generating contact forces by the astronauts and cosmonauts to navigate and propel themselves throughout the space station. Bones become biomechanically compromised when exposed to reduced forces and weightlessness (Zernicke et al., 1990). Therefore, the metabolic set point for bone homeostasis in an adult may depend on a bone’s location in the body, and its regular and systematic exposure to exercise-related ground reaction or contact forces. Osteocytes appear to play a role in skeletal regulation and may serve mechanosensory functions in bone (Plotkin and Bellido, 2016). In space or microgravity, the integrity of osteoblasts is compromised, while osteoclasts appear to continue to function (Nabavi et al., 2011). This binary system of our skeleton may be uniquely regulated compared to other systems, and therefore, the 1g environment of Earth has led to specific evolutionary characteristics that transcend the simple use it or lose it paradigm, and thus, the need for and the impact of exercise and loading on this tissue may be essential for bone health (Grimm et al., 2016).

During a period of rapid bone growth, however, there may be loading-independent aspects to bone. That is, during growth and maturation, if a knee is immobilized by pinning in flexion, the tibia continues to grow while muscles atrophy and ligaments of the knee cease to grow and mature (Hart et al., 2002). In a rabbit model, under rigid immobilization of the knee, the tibia continued to grow at a rapid pace. When the pin was subsequently removed, the knee joint could not be extended or opened due to bone overgrowth. The extent of this dysfunction gradually diminished when the immobilization was initiated as the age of the rabbits was increased from 3 to 6 to 8 weeks (Hart et al., 2002). In this period of rapid growth by the tibia, growth continued in the absence of mechanical loading, and thus, some biological factors may override the mechanical aspects during rapid growth. It also implies that bone growth in this situation was likely the driver of growth even in the absence of the ground reaction force stimuli, and the muscles, ligaments, and likely the tendons and muscles were responding to the growth of the bone rather than progressing in a co-stimulant manner. Whether support for this concept may be associated with the second phase of accelerated growth following onset of puberty remains unclear, as this phase is complicated by the presence of sex hormones, which can exert their own impact on muscles, bone, cardiovascular system, and cognitive functioning. However, once skeletal maturity has been achieved and an integrated set point for these systems is established, homeostasis and adaptations within a physiological window are likely regulated by use and mechanical loading (e.g., exercise, ground reaction and musculoskeletal forces, and daily physical activities on Earth).

With regard to the cardiovascular system changes in space or following exposure to microgravity, it is not clear whether the changes are due to a primary effect on the cells of the system or a secondary consequence of other alterations, such as changes to fluid regulation. It is clear, however, that the system continues to function well in spite of the changes associated with space flight as the incidence of cardiovascular events in space is very low. Therefore, many aspects of how and why the changes to the cardiovascular system occur remain to be elucidated. Nonetheless, it is well-documented that exercising in space (e.g., International Space Station) can be performed effectively and with many benefits (Macias et al., 2005).

Obviously, bone, muscles, most joint tissues, and the cardiovascular system all are innervated, and some of the above regulation may be associated with neural activity. Neuromuscular control and neuroRegulation of the cardiovascular system are well recognized, and the neural input into bone regulation is also supported (Elefteriou, 2005; Masi, 2012; Dimitri and Rosen, 2017). As bone and muscle are vascularized, there are likely essential interfaces among the systems affected by exposure to microgravity. In many tissues, the neural elements parallel the microvascularity, and the microvascular elements are likely linked to other cells in a tissue in a paracrine fashion, but the relative roles of elements and their responses to microgravity outside of the 1g environment in which they develop remain to be clarified (Grabherr and Mast, 2010). As these elements appear to work as integrated units to maintain functionality, the units likely require repeated and systematic loading to maintain their integrated function.

## Responses to Spaceflight and Microgravity Represent an Accelerated Form of Aging

Many of the physiological systems most affected by space flight and exposure to microgravity are those that are frequently and adversely affected by aging processes and associated increased incidence of diseases (e.g., osteoporosis, sarcopenia, cardiovascular diseases, and cognition decline). This similarity between responses to space/microgravity and aging was noted by Ray (1991), but many details regarding potential interrelations remain to be elucidated (Vernikos and Schneider, 2010). As we noted, these same systems, or disease entities, can be impacted by exercise, and as such, exercise is often viewed as “medicine” in that context. By extension, exercise via reinforcement of these systems designed to allow humans to operate in a 1g environment—before overt disease onset—would likely be beneficial to prevent the age-associated loss of system integrity leading to overt disease. As most astronauts have been younger than 50 years of age, they have been very healthy, but mature adults. The responses to space and microgravity are specific to the individual in the extent of the changes that occur, and it is not known whether there are family history linkages to specific relevant diseases in each astronaut. Furthermore, most astronauts to date have been males, and whether the responses of females are the same remains to be confirmed (Ploutz-Snyder et al., 2014). Essentially, it is unknown why the individual astronaut’s variations in response pattern exist. The changes observed and their extent may or may not be superimposed on specific genetic or epigenetic backgrounds, but this is a highly likely consideration (Hart, 2018a). Thus, under a 1g environment, silent genetic alterations may not become evident until the loading environment of living on Earth is removed by space flight or becomes evident during aging via more sedentary lifestyles. Thus, exercise and constant reinforcement of these 1g operational systems may overcome, at least in part, several genetic risk factors for disease development. As such, exercise and reinforcement of the relevant physiological systems may be particularly important during the last third of life for most individuals.

Based on the existing knowledge, much is known about **how** these systems may be regulated under Earth conditions, but we do not know a lot about **why** they are regulated in that manner, nor do we fully understand the impact of genetic and epigenetic variables on the integrity of these systems in the context of our 1g environment. Therefore, detailed analyses of individual-specific responses to microgravity and 1g during maturation and aging could provide new insights into the **why** the systems are regulated as they appear to be and identify new targets to focus on for new interventions. If these systems require constant reinforcement via exercise across the lifespan to thwart risk and maintain their integrity in *Homo sapiens*, then that could be a cost-effective approach to enhance healthy aging and mitigate health risks. Ideally that would entail having the types and doses of exercise optimized for specific physiological systems across the lifespan (Hart and Scott, 2012; Sjogaard et al., 2016) and personalized for each individual. In addition, optimal conditions will change during the different phases of life and cannot be considered constant. Similarly, what is required to maintain system integrity (e.g., threshold maintenance) versus optimizing system integrity (i.e., maximal impact) may also be different during growth, maturation, and senescence.

## The Distinctions Between Exercise Is Health Vs. Exercise Is Medicine

The preceding discussion aligns with other authors (Cheng and Mao, 2016; Sjogaard et al., 2016; Smith, 2016), who have highlighted that distinctions exist between “Exercise is Health” vs. “Exercise is Medicine.” Those distinctions are not static, but are dynamically influenced by multiple factors. Firstly, for those with chronic diseases or conditions, one distinction may depend on the status of the patient’s disease process when an exercise regimen is initiated. Those with early disease and modest tissue-altering pathology may respond better via interfering with the disease progression than patients that have more progressed pathology. In contrast, those with advanced disease, with associated pathological disruption of tissue integrity and more extensive epigenetic alterations, would potentially be less likely to exhibit a positive response to exercise than patients in early disease. Furthermore, the use of exercise in conjunction with suitable drugs or compounds, could provide additive or synergistic effects for the exercise component (Hart, 2018c). Secondly, if epigenetic alterations, and not just tissue disruptive pathology, are playing a critical role in the distinction between whether exercise is health vs. medicine, then it may be possible to reverse the epigenetic alterations using drugs or other interventions. While not being driven by a traditional disease process, it was noted that some of the epigenetic changes detected after a 1-year space flight were reversible after return to Earth^1^ (Garrett-Bakelman et al., 2019). Thus, some epigenetic alterations to the human genome are reversible once a return to the 1g environment is established. Therefore, the distinctions between “Exercise is Health vs. Medicine” may be malleable, and there may be opportunities to shift the effectiveness of exercise for those with chronic diseases back to that of the healthy individual via interventions, which impact epigenetic alterations associated with disease.

## Conclusion

If the proposed inverse relations are valid between the benefits of exercise and the effects of microgravity exposure on similar systems, then detailed mechanistic clues as to why exercise is health may be gleaned and could be effectively studied to elaborate new and critical insights. The most obvious conclusion is that our 1g-dependent systems require constant reinforcement to be optimized and maintained throughout life. Similar to mastering a sport or a musical instrument, practice makes perfect; these 1g-dependent systems may require regular and focused attention to maintain their integrity and effectiveness, at the levels of individual tissues and via central neural control mechanisms. Thus, the premise that “Exercise is Health” may be the preferred designation and have much of its basis in the maintenance of these 1g-dependent systems to prevent or inhibit their dysfunction or senescence (i.e., loss of system integrity with advancing age). As such, the “Exercise is Health” designation for impact on those who have not lost their health is a valid distinction from “Exercise is Medicine,” which applies to those who have lost their health in an impacted system that is altered by associated pathology (e.g., epigenetics, altered cell types, and/or different cell types) and its treatment. Thus, exercise is essential to life for humans on Earth and is intrinsic to optimized and sustained system integrity across a person’s lifespan.

Sedentary behavior and a lack of a minimal or reinforcing loading may allow for exercise/loading-inhibited otherwise “silent” mutations in our genomes to overcome such inhibition and contribute to senescence, and what we now label “aging related” declines in integrity. As such, a more apt designation for **exercise** is that it **is intrinsic to who we are,** and optimal exercise is essential to maintain and sustain integrated optimal functioning. The unique blend of independent and integrated systems influenced by exercise defines how humans evolved to thrive in Earth’s 1g environment. This perspective is supported by Pontzer (2019), who discussed the role of exercise in human physiology as *Homo sapiens* evolved from great-ape lineages. Detailed investigation of how these 1g-related systems work, where they are controlled, and how they are regulated (and inter-regulated) by exercise and mobility may provide unique and significant new clues for maintaining optimal health and mitigating risks for loss of health.

## Author Contributions

DH wrote the initial draft of the review, and then both authors contributed equally to its further development and finalization.

## Conflict of Interest

The authors declare that the research was conducted in the absence of any commercial or financial relationships that could be construed as a potential conflict of interest.

## Footnotes

1 https://www.whatisepigenetics.com/epigenetic-research-space-exploration/

## References

[B1] Ahuja Y. R. Sharma S. Mohan V. (2017). Cardiovascular diseases: interplay of epigenetics. *Clin. Exp. Hypertens.* 39 1–7. 10.1080/10641963.2016.1210627 28055289

[B2] Ando J. Kamiya A. (1996). Flow-dependent regulation of gene expression in vascular endothelial cells. *Jpn. Heart J.* 37 19–32. 10.1536/ihj.37.19 8632623

[B3] Armamento-Villareal R. Aguire L. Waters D. L. Napoli N. Qualls C. Villareal D. T. (2019). Effect of aerobic or resistance exercise, or both, on bone mineral density and bone metabolism in obese older adults while dieting: a randomized controlled trial. *J. Bone Mineral Res.* [Epub ahead of print]. 10.1002/jbmr.3905 PMC7064383 31797417

[B4] Ashe M. C. Khan K. M. (2004). Exercise prescription. *J. Am. Acad. Orthop. Surg.* 12 21–27. 14753794 10.5435/00124635-200401000-00004

[B5] Axsom J. E. Libonati J. R. (2019). Impact of parental exercise on epigenetic modifications inherited by offspring: a systematic review. *Physiol. Rep.* 7:e14287. 10.14814/phy2.14287 PMC6874781 31758667

[B6] Barha C. K. Galea L. A. Nagamatsu L. S. Erickson K. I. Liu-Ambrose T. (2017). Personalizing exercise recommendations for brain health: considerations and future directions. *Br. J. Sports Med.* 51 636–639. 10.1136/bjsports-2016-096710 27856411

[B7] Barha C.K. Hsu C.-L. ten Brinke L. Liu-Ambrose T. (2019). Biological sex: a potential moderator of physical activity efficacy on brain health. *Front. Aging Neurosci*. 11:329. 10.3389/fnagi.2019.00329 31866852 PMC6908464

[B8] Bays H. Scinta W. (2015). Adiposopathy and epigenetics: an introduction to obesity as a transgenerational disease. *Curr. Med. Res. Opin.* 31 2059–2069. 10.1185/03007995.2015.1087983 26331354

[B9] Boisseau M. R. (2005). Roles of mechanical blood forces in vascular diseases: a clinical overview. *Clin. Hemorheol Microcir.* 33 201–207. 16215286

[B10] Brunet A. Berger S. L. (2014). Epigenetics of aging and aging-related disease. *J. Gerontol. A Biol. Sci. Med. Sci.* 69(Suppl. 1), S17–S20. 24833581 10.1093/gerona/glu042 PMC4022130

[B11] Burns J. M. Cronk B. B. Anderson H. S. (2008). Cardiorespiratory fitness and brain atrophy in early Alzheimer’s disease. *Neurology* 71 210–216. 10.1212/01.wnl.0000317094.86209.cb 18625967 PMC2657657

[B12] Carter H. N. Chen C. C. Hood D. A. (2015). Mitochondria, muscle health, and exercise with advancing age. *Physiology (Bethesda)* 30 208–223. 10.1152/physiol.00039.2014 25933821

[B13] Cheng S. Mao L. (2016). Physical activity continuum throughout the lifespan: is exercise medicine or what? *J. Sport Health Sci.* 5 127–128. 10.1016/j.jshs.2016.03.005 30356514 PMC6188714

[B14] Colcombe S. J. Kramer A. F. (2003). Fitness effects on the cognitive function of older adults: a meta-analytic study. *Psychol. Sci.* 14 125–130. 10.1111/1467-9280.t01-1-01430 12661673

[B15] Collins K. H. Herzog W. Reimer R. A. Reno C. R. Heard B. J. Hart D. A. (2018). Pro-inflammatory vitreous humour alterations as a result of high fat-high sucrose diet-induced obesity in a rat model. *Inflamm. Res.* 67 139–146. 10.1007/s00011-017-1102-y 29075814

[B16] Collins K. H. Paul H. A. Hart D. A. Reimer R. A. Smith I. C. Rios J. L. (2016). A high-fat high-sucrose diet rapidly alters muscle integrity, inflammation and gut microbiota in male rats. *Sci. Rep.* 6:37278. 10.1038/srep37278 PMC5112513 27853291

[B17] Cribbs A. Feldmann M. Oppermann U. (2015). Towards understanding of the role of DNA methylation in rheumatoid arthritis: therapeutic and diagnostic implications. *Ther. Adv. Musculoskelet Dis.* 7 206–219. 10.1177/1759720x15598307 26425149 PMC4572365

[B18] Dao E. Barha C. K. Best J. R. Hsiung G. Y. Tam R. Liu-Ambrose T. (2019). The effect of aerobic exercise on white matter hyperintensity progression may vary by sex. *Can. J. Aging* 38 236–244. 10.1017/s0714980818000582 30867079

[B19] Denham J. (2018). Exercise and epigenetic inheritance of disease risk. *Acta Physiol.* 222 1–20. 10.1111/apha.12881 28371392

[B20] Desiderio A. Speinelli R. Ciccoarelli M. Nigro C. Miele C. Bequinot F. (2016). Epigenetics: spotlight on type 2 diabetes and obesity. *J. Endocrinol. Invest.* 39 1095–1103. 10.1007/s40618-016-0473-1 27180180

[B21] DeSouza M. J. Nattiv A. Joy E. Misra M. Williams N. I. Mallinson R. J. (2014). Female athlete triad coalition consensus statement on treatment and return to play of the female athlete triad. *Br. J. Sports Med.* 48 289–309. 25014387 10.1249/JSR.0000000000000077

[B22] Dimitri P. Rosen C. (2017). The central nervous system and bone metabolism: an evolving story. *Calcif. Tissue Int.* 100 478–485. 10.1007/s00223-016-0179-6 27501818

[B23] Eckel J. (2019). Myokines in metabolic homeostasis and diabetes. *Diabetologia* 62 1523–1528. 10.1007/s00125-019-4927-9 31263909

[B24] Elefteriou F. (2005). Neuronal signaling and the regulation of bone remodeling. *Cell Mol. Life. Sci.* 62 2339–2349. 10.1007/s00018-005-5175-3 16132233 PMC11139174

[B25] Erickson K. I. Kramer A. F. (2009). Aerobic exercise effects on cognitive and neural plasticity in older adults. *Br. J. Sports Med.* 43 22–24. 10.1136/bjsm.2008.052498 18927158 PMC2853472

[B26] Friedl K. E. Evans R. K. Moran D. S. (2008). Stress fracture and military medical readiness: bridging basic and applied research. *Med. Sci. Sports Exerc.* 40 S609–S622. 18849874 10.1249/MSS.0b013e3181892d53

[B27] Garrett-Bakelman F. E. Darshi M. Green S. J. Gur R. C. Lin L. (2019). The NASA twins study: a multidimensional analysis of a year-long human spaceflight. *Science* 364 1–21. 10.1126/science.aau8650 30975860 PMC7580864

[B28] Ghayor C. Weber F. E. (2016). Epigenetic regulation of bone remodeling and its impacts on osteoporosis. *Int. J. Mol. Sci.* 17 E1446. 10.3390/ijms17091446 PMC5037725 27598138

[B29] Ghosh A. K. Rai R. Flevaris P. Vaughan D. E. (2017). Epigenetics in reactive and reparative cardiac fibrogenesis: the promise of epigenetic therapy. *J. Cell. Physiol.* 232 1941–1956. 10.1002/jcp.25699 27883184

[B30] Giudice J. Taylor J. M. (2017). Muscle as a paracrine and endocrine organ. *Curr. Opin. Pharmacol.* 34 49–55. 10.1016/j.coph.2017.05.005 28605657 PMC5808999

[B31] Grabherr L. Mast F. W. (2010). Effects of microgravity on cognition: the case of mental imagery. *J. Vestib. Res.* 20 53–60. 10.3233/ves-2010-0364 20555167

[B32] Grazioli E. Dimauro I. Mercatelli N. Wang G. Pitsiladis Y. DiLuigi L. (2017). Physical activity in the prevention of human diseases: role of epigenetic modifications. *BMC Genomics* 18(Suppl. 8):802. 10.1186/s12864-017-4193-5 29143608 PMC5688489

[B33] Grimm D. Grosse J. Wehland M. Mann V. Presland J. E. Sundaresan A. (2016). The impact of microgravity on bone in humans. *Bone* 87 44–56. 10.1016/j.bone.2015.12.057 27032715

[B34] Hart D. A. (2018a). Are we learning as much as possible from spaceflight to better understand health and risks to health on Earth, as well as in space? *J. Biomed. Sci. Eng.* 11 109–118. 10.4236/jbise.2018.116010

[B35] Hart D. A. (2018b). Evidence for a potential “knee-eye-brain” axis involved in mobility and navigation control. *J. Biomed. Sci. Eng.* 11 37–44. 10.4236/jbise.2018.113004

[B36] Hart D. A. (2018c). Would adding low doses of lithium salts and/or prebiotic fibre interventions to an effective exercise protocol further enhance retention of cognitive integrity? Potential for preventing loss of cognition with aging using combinations of low cost regimens. *J. Biomed. Sci. Eng.* 11 1–9. 10.4236/jbise.2018.111001

[B37] Hart D. A. Herzog W. Reimer R. A. Rios J. L. Collins K. H. (2019). Obesity and impact on host systems effecting mobility and potentially, on fidelity of navigation through the environment. *Eur. Med. J.* 4 63–70.

[B38] Hart D. A. Natsu-ume T. Sciore P. Tasevski V. Frank C. B. Shrive N. G. (2002). Mechanobiology: similarities and differences between in vivo and in vitro analysis at the functional and molecular levels. *Recent Res. Dev. Biophys. Biochem.* 2 153–177.

[B39] Hart D. A. Scott A. (2012). Getting the dose right when prescribing exercise for connective tissue conditions: the yin and the yang of tissue homeostasis. *Br. J. Sports Med.* 46 696–698. 10.1136/bjsports-2011-090083 21908882

[B40] Heyn P. Abreu B. C. Ottenbacher K. J. (2004). The effects of exercise training on elderly persons with cognitive impairment and dementia: a meta-analysis. *Arch. Phys. Med. Rehab.* 84 1694–1704. 10.1016/j.apmr.2004.03.019 15468033

[B41] Hills A. P. Street S. J. Byrne N. M. (2015). Physical activity and health: “what is old is new again”. *Adv. Food Nutr. Res.* 75 77–95. 26319905 10.1016/bs.afnr.2015.06.001

[B42] Hughson R. L. Robertson A. D. Arbeille P. Shoemaker J. K. Rush J. W. Fraser K. S. (2016). Increased postflight carotid artery stiffness and inflight insulin resistance resulting from 6-mo spaceflight in male and female astronauts. *Am. J. Physiol. Heart Circ. Physiol.* 310 H628–H638. 26747504 10.1152/ajpheart.00802.2015

[B43] Hughson R. L. Shoemaker J. K. (2015). Autonomic responses to exercise: deconditioning/inactivity. *Auton. Neurosci.* 188 132–135. 10.1016/j.autneu.2014.10.012 25458429

[B44] Johnson B. D. Mather K. J. Wallace J. P. (2011). Mechanotransduction of shear in the endothelium: basic studies and clinical implications. *Vasc. Med.* 16 365–377. 10.1177/1358863x11422109 22003002

[B45] Jones H. A. Cowan D. N. Knapik J. J. (1994). Exercise, training, and injuries. *Sports Med.* 18 202–214. 10.2165/00007256-199418030-00005 7809556

[B46] Joseph A. M. Collins C. L. Henke N. M. Yard E. E. Fields S. K. Comstock R. P. (2013). A multisport epidemiologic comparison of anterior cruciate ligament injuries in high school athletics. *J. Athletic Train.* 48 810–817. 10.4085/1062-6050-48.6.03 24143905 PMC3867093

[B47] Karnik S. Kanekar A. (2012). Childhood obesity: a global public health crisis. *Int. J. Prev. Med.* 3 1–7. 22506094 PMC3278864

[B48] Koida G. Hambrecht R. (2005). Molecular mechanisms of vascular adaptations to exercise. Physical activity as an effective antioxidant therapy? *Cardiovasc. Res.* 67 187–197. 10.1016/j.cardiores.2005.04.032 15935334

[B49] Kos O. Hughson R. L. Hart D. A. Clement G. Frings-Meuthen P. Linnarsson D. (2014). Elevated serum soluble CD200 and CD200R as surrogate markers of bone loss under bed rest conditions. *Bone* 60 33–40. 10.1016/j.bone.2013.12.004 24333170

[B50] Kramer A. F. Hahn S. Cohen N. J. (1999). Aging, fitness, and neurocognitive function. *Nature* 400 418–419. 10440369 10.1038/22682

[B51] Kraus E. Tenforde A. S. Nattiv A. Sainani K. L. Kussman A. Deakin-Roche M. (2019). Bone stress injuries in male distance runners: higher modified female athlete triad cumulative risk assessment scores predict increased rates of injury. *Br. J. Sports Med.* 53 237–242. 10.1136/bjsports-2018-099861 30580252

[B52] Kydd A. S. Tsao H. Reno C. Hart D. A. (2007). Impact of age, systemic glucocorticoids and progressive osteoarthritis on specific mRNA levels in different areas of the rabbit cornea. *Cornea* 26 352–361. 10.1097/ico.0b013e318033a534 17413965

[B53] Ling C. Ronn T. (2019). Epigenetics in human obesity and type 2 diabetes. *Cell Metab.* 29 1028–1044. 10.1016/j.cmet.2019.03.009 30982733 PMC6509280

[B54] Liu-Ambrose T. Donaldson M. G. (2009). Exercise and cognition in older adults: is there a role for resistance training programmes? *Br. J. Sports Med.* 43 25–27. 10.1136/bjsm.2008.055616 19019904 PMC5298919

[B55] Ma C. L. Ma X. T. Wang J. J. Liu H. Chen Y. F. Yamng Y. (2016). Physical exercise induces hippocampal neurogenesis and prevents cognitive decline. *Behav. Brain Res.* 317 332–339. 10.1016/j.bbr.2016.09.067 27702635

[B56] Macias B. R. Groppo E. R. Eastlack R. K. Waterpaugh D. E. Lee S. M. Schneider S. M. (2005). Space exercise and Earth benefits. *Curr. Pharm. Biotechnol.* 6 305–317. 10.2174/1389201054553653 16101469

[B57] Majima T. Marchuk L. L. Sciore P. Shrive N. G. Frank C. B. Hart D. A. (2000). Compressive compared with tensile loading of medial collateral ligament scar in vitro uniquely influences mRNA levels for aggrecan, collagen type II, and collagenase. *J. Orthop. Res.* 18 524–531. 10.1002/jor.1100180403 11052487

[B58] Masi L. (2012). Crosstalk between the brain and bone. *Clin. Cases Miner. Bone Metab.* 9 13–16. 22783328 PMC3392669

[B59] Moreira L. D. Oliveira M. L. Lirani-Galvao A. P. Marin-Mio R. V. Santos R. N. Lazaretti-Castro M. (2014). Physical exercise and osteoporosis: effects of different types of exercises on bone and physical function of postmenopausal women. *Arq. Bras. Endocrinol. Metabol.* 58 514–522. 10.1590/0004-2730000003374 25166042

[B60] Morey-Holton E. R. (2003). “The impact of gravity on life,” in *Evolution on Planet Earth: Impact of the Physical Environment*, eds Rothchild L. J. Lister A. M. (Amsterdam: Elsevier), 143–159. 10.1016/b978-012598655-7/50036-7

[B61] Muka T. Nano J. Voortman I. Braun K. V. E. Ligthart S. Stranges S. (2016). The role of global and regional DNA methylation and histone modifications in glycemic traits and type 2 diabetes: a systematic review. *Nutr. Metab. Cardiovasc. Dis.* 26 553–566. 10.1016/j.numecd.2016.04.002 27146363

[B62] Nabavi N. Khandani A. Camirand A. Harrison R. E. (2011). Effects of microgravity on osteoclast bone resorption and osteoblast cytoskeletal organization and adhesion. *Bone* 49 965–974. 10.1016/j.bone.2011.07.036 21839189

[B63] Natsu-ume T. Majima T. Reno C. Shrive N. G. Frank C. B. Hart D. A. (2005). Menisci of the rabbit knee require mechanical loading to maintain homeostasis: cyclic hydrostatic compression in vitro prevents derepression of catabolic genes. *J. Orthop. Sci.* 10 396–405. 10.1007/s00776-005-0912-x 16075173

[B64] Nielsen S. Pedersen B. K. (2008). Skeletal muscle as an immunogenic organ. *Curr. Opin. Pharmacol.* 8 346–351. 10.1016/j.coph.2008.02.005 18417420

[B65] Ohira T. Kawano F. Ohira T. Goto K. Ohira Y. (2015). Responses of skeletal muscles to gravitational unloading and/or reloading. *J. Phys. Sci.* 65 293–310. 10.1007/s12576-015-0375-6 25850921 PMC10717835

[B66] Pagiatakis C. Musolino E. Gornati R. Bernardini G. Papait R. (2019). Epigenetics of aging and disease. A brief overview. *Aging Clin. Exp. Res.* [Epub ahead of print]. 10.1007/s40520-019-01430-0 PMC8084772 31811572

[B67] Pal S. Tyler J. K. (2016). Epigenetics and aging. *Sci. Adv.* 2 e1600584. 10.1126/sciadv.1600584 PMC4966880 27482540

[B68] Paul H. A. Collins K. H. Nicolucci A. C. Urbanski S. J. Hart D. A. Vogel H. J. (2019). Maternal prebiotic supplementation reduces fatty liver development in offspring through altered microbial and metabolomics profiles in rats. *FASEB J.* 33 5153–5167. 10.96/fj.20180‘551R 30629464

[B69] Pedersen B. K. (2011). Muscles and their myokines. *J. Exp. Biol.* 214 337–346. 10.1242/jeb.048074 21177953

[B70] Pedersen B. K. (2017). Anti-inflammatory effects of exercise: role in diabetes and cardiovascular disease. *Eur. J. Clin. Invest.* 47 600–611. 10.1111/eci.12781 28722106

[B71] Pedersen B. K. Febbraio M. A. (2012). Muscles, exercise, and obesity: skeletal muscles as a secretory organ. *Nat. Rev. Endocrinol.* 8 457–465. 10.1038/nrendo.2012.49 22473333

[B72] Pedersen B. K. Saltin B. (2015). Exercise is medicine—evidence for prescribing exercise as therapy in 26 different chronic diseases. *Scand. J. Med. Sci. Sports* 25(Suppl. 3), 1–7. 10.1111/sms.12581 26606383

[B73] Pigeyre M. Yazdi F. T. Kaur Y. Myere D. (2016). Recent progress in genetics, epigenetics, and metagenomics unveils the pathophysiology of human obesity. *Clin. Sci. (Lond.)* 130 943–986. 10.1042/cs20160136 27154742

[B74] Piletic K. Kunei T. (2016). MicroRNA epigenetic signatures in human disease. *Arch. Toxicol.* 90 2405–2419. 10.1007/s00204-016-1815-7 27557899

[B75] Plotkin L. I. Bellido T. (2016). Osteocytic signalling pathways as therapeutic targets for bone fragility. *Nat. Rev. Endocrinol.* 12 593–605. 10.1038/nrendo.2016.71 27230951 PMC6124897

[B76] Ploutz-Snyder L. Bloomfield S. Smith S. M. Hunter S. K. Templeton K. Bemben D. (2014). Effects of sex and gender on adaptation to space: musculoskeletal health. *J. Women’s Health (Larchmont.)* 23 963–966. 10.1089/jwh.2014.4910 25401942 PMC4235589

[B77] Pontzer H. (2019). Evolved to exercise. *Sci. Am.* 320 23–29. 10.1038/scientificamerican0119-22 38918920

[B78] Qazi T. J. Quan Z. Mir A. Qing H. (2018). Epigenetics in Alzheimer’s disease: perspective of DNA methylation. *Mol. Neurobiol.* 55 1026–1044. 10.1007/s12035-016-0357-6 28092081

[B79] Raghuraman S. Donkin I. Versteyhe S. Barres R. Simar D. (2016). The emerging role of epigenetics in inflammation and immunometabolism. *Trends Endocrinol. Metab.* 27 782–795. 10.1016/j.tem.2016.06.008 27444065

[B80] Raman S. FitzGerald U. Murphy J. M. (2018). Interplay of inflammatory mediators with epigenetics and cartilage modifications in osteoarthritis. *Front. Bioeng. Biotechnol.* 6:22. 10.3389/fbioe.2018.00022 29594113 PMC5861204

[B81] Ray E. A. (1991). Introduction: are aging and space effects similar? *Exp. Gerontol.* 26 123–129. 10.1016/0531-5565(91)90002-4 1680731

[B82] Ribeiro P. A. Boidin M. Juneau M. Nigam A. Gayda M. (2016). High-intensity interval training in patients with coronary heart disease: prescription models and perspectives. *Ann. Phys. Rehabil. Med.* 60 50–57. 10.1016/j.rehab.2016.04.004 27346629

[B83] Rios J. L. Bomhof M. Reimer R. A. Hart D. A. Collins K. H. Herzog W. (2019). Protective effect of prebiotic and exercise intervention on knee health in a rat model of diet-induced obesity. *Sci. Rep.* 9:3893. 10.1038/s41598-019-40601-x PMC6405910 30846801

[B84] Risberg M. A. Lewek M. Snyder-Mackler L. (2004). A systematic review of evidence for anterior cruciate ligament rehabilitation: how much and what type? *Phys. Ther. Sport* 5 125–145. 10.1016/j.ptsp.2004.02.003

[B85] Ross L. M. Porter R. R. Durstine L. J. (2016). High-intensity interval training (HIIT) for patients with chronic diseases. *J. Sport Health Sci.* 5 139–144. 10.1016/j.jshs.2016.04.005 30356536 PMC6188712

[B86] Sallis R. E. (2009). Exercise is medicine and physicians need to prescribe it. *Br. J. Sports Med.* 43 3–4. 10.1136/bjsm.2008.054825 18971243

[B87] Scott A. Khan K. M. Duronio V. Hart D. A. (2008). Mechanotransduction in human bone: in vitro cellular physiology that underpins bone changes with exercise. *Sports Med.* 38 139–160. 10.2165/00007256-200838020-00004 18201116 PMC3951486

[B88] Sharples A. P. Stewart C. E. Seaborne R. A. (2016). Does skeletal muscle have an “epi” memory? The role of epigenetics in nutritional programing, metabolic disease, aging, and exercise. *Aging Cell* 15 603–616. 10.1111/acel.12486 27102569 PMC4933662

[B89] Sjogaard G. Christensen J. R. Justesen J. B. Murray M. Dalager T. Fredslund G. H. (2016). Exercise is more than medicine: the working age population’s well-being and productivity. *J. Sports Health Sci.* 5 159–165. 10.1016/j.jshs.2016.04.004 30356522 PMC6188718

[B90] Smith A. (2016). Exercise is recreation not medicine. *J. Sport Health Sci.* 5 129–134. 30356503 10.1016/j.jshs.2016.03.002 PMC6188606

[B91] Sparks L. M. (2017). Exercise training response heterogeneity: physiological and molecular insights. *Diabetologia* 60 2309–2336. 10.1007/s00125-017-4461-6 29032385

[B92] Sqalla G. Iovene B. Calvello M. Ori M. Varone F. Richeldi L. (2018). Idiopathic pulmonary fibrosis: pathogenesis and management. *Respir Res.* 19:32. 10.1186/s12931-018-0730-2 PMC5824456 29471816

[B93] Tari A. R. Nauman J. Zisko N. Skjellengrind H. K. Bosnes I. Bergh S. (2019). Temporal changes in cardiorespiratory fitness and risk of dementia incidence and mortality: a population-based prospective cohort study. *Lancet* 4 e565–e571. 31677775 10.1016/S2468-2667(19)30183-5

[B94] Tenforde A. S. Carlson J. L. Chang A. Sainani K. L. Shultz R. Kim J. H. (2016). Association of the Female Athlete Triad risk assessment stratification to the development of bone stress injuries in collegiate athletes. *Am. J. Sports Med.* 45 302–310. 10.1177/0363546516676262 28038316

[B95] Tenforde A. S. Carlson J. L. Sainani K. L. O. Chang A. O. Kim J. H. Golden N. H. (2018). Sport and Triad risk factors influence bone mineral density in collegiate athletes. *Med. Sci. Sports Exerc.* 50 2536–2543. 10.1249/mss.0000000000001711 29975299

[B96] Trudel G. Payne M. Madler B. Ramachandran N. Lecompte M. Wade C. (2009). Bone marrow fat accumulation after 60 days of bed rest persisted 1 year after activities were resumed along with hemopoietic stimulation: the women international space simulation for exploration study. *J. Appl. Physiol.* 107 540–548. 10.1152/japplphysiol.91530.2008 19478189

[B97] Van Meurs J. B. (2017). Osteoarthritis year in review 2016: genetics, genomics and epigenetics. *Osteoarthritis Cartilage* 25 181–189. 10.1016/j.joca.2016.11.011 28100422

[B98] Vernikos J. Schneider V. S. (2010). Space, gravity, and the physiology of aging—parallel or convergent disciplines: a mini-review. *Gerontology* 56 157–166. 10.1159/000252852 19851058

[B99] Way K. L. Hackett D. A. Baker M. K. Johnson N. A. (2016). The effect of regular exercise on insulin sensitivity in type 2 diabetes mellitus: a systematic review and meta-analysis. *Diabetes Metab. J.* 40 253–271. 27535644 10.4093/dmj.2016.40.4.253 PMC4995180

[B100] Widmann M. Niess A. M. Munz B. (2019). Physical exercise and epigenetic modifications in skeletal muscle. *Sports Med.* 49 509–523. 10.1007/s40279-019-01070-4 30778851

[B101] Won J. Alfini A. J. Weiss L. R. Michelson C. S. (2019). Semantic memory activation after acute exercise in healthy older adults. *J. Int. Neuropsychol. Soc.* 25 557–568. 10.1017/s1355617719000171 31018875

[B102] Xu W. Wang F. Yu Z. Xin F. (2016). Epigenetics and cellular metabolism. *Genet. Epigenet.* 8 43–51. 27695375 10.4137/GEG.S32160 PMC5038610

[B103] Zernicke R. F. Garhammer J. J. Jobe F. W. (1977). Human patellar tendon rupture: a kinetic analysis. *J. Bone Joint Surg.* 59A 179–183. 845201

[B104] Zernicke R. F. Vailas A. C. Salem G. J. (1990). Biomechanical response of bone to weightlessness. *Exerc. Sport Sci. Rev.* 18 167–192. 2192892

